# Impairments following COVID-19 infection: manifestations and investigations of related factors

**DOI:** 10.1038/s41598-023-33810-y

**Published:** 2023-04-21

**Authors:** Farzaneh Badinlou, David Forsström, Markus Jansson-Fröjmark, Tamar Abzhandadze, Tobias Lundgren

**Affiliations:** 1 Department of Clinical Neuroscience, Centre for Psychiatry Research, Karolinska Institute, Stockholm Health Care Services, Region Stockholm, Stockholm, Sweden; 2 Medical Unit Medical Psychology, Women’s Health and Allied Health Professionals Theme, Karolinska University Hospital, Solna, Sweden; 3 Department of Psychology, Stockholm University, Stockholm, Sweden; 4 Department of Clinical Neuroscience, Institute of Neuroscience and Physiology, The Sahlgrenska Academy, University of Gothenburg, Gothenburg, Sweden; 5 Department of Occupational Therapy and Physiotherapy, Sahlgrenska University Hospital, Gothenburg, Sweden

**Keywords:** Psychology, Public health

## Abstract

The COVID-19 pandemic had a major global impact on the health and wellbeing for many individuals. Even though the infection rates have gone down due to the availability of vaccines, the consequences of the disease are still present due to persistent symptoms among individuals. The aim of the current study was to map long‐term impairments in individuals infected with COVID-19 by applying the framework of the World Health Organization’s International Classification of Functioning, Disability and Health (ICF) and also investigate the factors related to the context of an individual’s life influencing the impairments. A web-survey that targeted individuals that had been infected with COVID-19 was used. The survey included a range of measures covering contextual factors and factors related to body functions and structures and post-COVID impairments. A total of 501 individuals were included in the study (with a mean age of 47.6 years). 96% of the respondents reported at least one moderate-to-severe impairment due to COVID-19 infection and the most frequent one was fatigue. In that, 79.6% and 86.9% of the study sample reported moderate-to-severe brain fatigue and tiredness or lack of energy, respectively. Severity of COVID-19 infection appeared as the strongest risk factor for post-COVID impairments. Based on the results, interventions can be tailored to help individuals with post-COVID-19 condition. This could be one way lessening the effect of COVID-19 on health care and society as a whole.

## Introduction

Emerging evidence has shown that a great number of individuals infected with COVID-19 continue to suffer from persistent symptoms after the onset of illness or hospitalization^1–6^. Early studies showed that more than 30% of the individuals infected with COVID-19 experienced long-term SARS-CoV-2 infection–associated symptoms after the acute phase^7,8^. According to recent meta-analyses based on data from different studies from different countries, the global prevalence of persistent symptoms was higher. In that, nearly half of the people infected with SARS CoV-2 infection experienced a wide range of impairments and difficulties months and even one year after COVID-19 infection^3,9,10^.

Several studies have investigated long-term symptoms of COVID-19 weeks or months after the acute phase of the disease mainly symptoms lasting more than 12 weeks. Studies mainly focusing on individuals after recovery or discharge from the hospital have reported a wide range of persistent symptoms, such as fatigue, dyspnea, anosmia, palpitations, insomnia, chest pain, headache, cough, mental health problems, and cognitive impairments, in different populations in different countries^5,11–15^. However, a significant proportion of individuals infected with the SARS-CoV-2 experienced mild-to-moderate symptoms and mostly recovered without requiring special treatment or/and hospitalization^16^. A longitudinal study showed that shortness of breath, anosmia, ageusia, and fatigue are the most common symptoms in non-hospitalized patients 7 months after the onset of disease^11^. In an overview, post-infection COVID-19 patients showed persisting symptoms involving multiple-organ impairment, such as impaired respiratory function^17^, cognitive functions^18^, cardiovascular function^19^, and muscular functions^20^. A higher probability of persistent symptoms in the general population are associated with being women, older age, obesity, smoking or vaping, hospitalization, admitted to intensive care, and deprivation^6,21^. In addition, vaccination status and COVID-19 reinfection may be associated with post-COVID complaints^22–25^. In that, vaccinated against COVID-19 was associated with decreasing in post-COVID complaints by reducing risk of reinfection and providing additional immunological protection^23–26^. Post-COVID entails an array of symptoms that has not been thoroughly systematized. To take more effective actions to understand and evaluate post-COVID-19 complaints, the bio-psych-social framework of the WHO International Classification of Functioning, Disability and Health (ICF) framework was recommended to use^21,27^. The ICF framework represents the assessment of body functions and structures, activities and participation, and environmental and personal factors^28^. The ICF framework was used in the meta-analysis studies in order to report physical and mental health post-COVID-19 complaints^21,27^. However, there is a limited number of scientific publications that have used the ICF framework directly to assess and classify post-COVID symptoms. One example is the Clinical Functioning Information Tool (ClinFIT COVID- 19), developed by the International Society of Physical and Rehabilitation Medicine (ISPRM) used for the assessment and reporting of functioning in COVID-19 patients/survivors in acute, post-acute, and long-term care settings. ClinFIT COVID- 19 consists of 16 categories/codes including body functions (9 codes), activities and participations (6 codes), and body structures (one code)^29^. However, the ClinFIT COVID- 19 has some limitations, such as lack of operationalizations category on the final ICF category list into items/questions, not considering scoring options for rating the patient's status in each item, and not considering the effects of personal and environmental factors. Another example is the Functional Compass COVID-19 questionnaire, developed as a self-report instrument in order to determine impairments in body functions and activities following mild COVID-19 infection. This questionnaire consists of items covering central nervous (CNS) functions (9 codes), somatic body functions (14 codes), and activities and participations (15 codes)^30^. The most impaired CNS functions were fatigability and energy and drive function, the most impaired somatic body functions were impairments in respiratory and impaired muscle power, and the most impaired activity was impairment in handling stress and other psychological demands. However, this questionnaire has also some limitations, such as the small number of participants and not considering personal and environmental factors**.** There is therefore a need to develop the ICF categories in relation to post-COVID impairments and examine the influence of factors that might contribute to its severity in a comprehensive way.

Since the beginning of COVID-19 pandemic, over two million confirmed cases, including more than 23,000 deaths, have been reported in Sweden^31^. A population-based cohort study in Sweden showed that 2% of all COVID-19 cases have been diagnosed with post-COVID condition mostly individuals who had not been hospitalized for COVID-19^32^. However, a range of post-COVID condition was estimated 9–81% in a systematic review depending on differences in the study populations and methods^9^. The clinical presentations of post-COVID complications vary and it is not clear how many people can have post-COVID impairments but not diagnosed with post-COVID conditions. To summarize, long‐term impacts of COVID ‐19 infection can be multi-dimensional when it comes to health-related problems and disability^9,21^. Therefore, the primary aim of this study was to map long‐term impairments in individuals infected with COVID-19 by applying the framework of the World Health Organization’s International Classification of Functioning, Disability and Health^28^. To further enhance the knowledge about post-COVID impairments, the secondary aim of the study was to address the identification of contextual factors and factors related to body functions and structures associated with impairments following COVID-19 infection. According to the ICF, contextual factors refer to the context of an individual’s life and comprise two components: personal factors and environmental factors. Personal factors are defined as the particular background of an individual’s life and living and comprise features of the individual that are not part of a health condition or health states. Environmental factors are defined as the physical, social, and attitudinal environment in which people live and conduct their lives^28^.

## Methods

### Study design and participants

The study is designed as a cross-sectional study based on the data from self-reported questionnaires**.** By considering the number of COVID-19 cases in Sweden and the average sample sizes utilized in similar studies investigating post-COVID complaints, the sample size for the current study was estimated approximately 519 patients. Inclusion criteria were: (1) infected with COVID-19; (2) age (≥ 18 years); (3) being a resident of Sweden; (4) fluent in Swedish and access to the internet in order to complete the online-survey. A total 501 participants representing all counties in Sweden was included in the current study through convenience sampling method. Data were collected between the 23th of February and the 1th of April 2022.

### Procedures

Participants were recruited through convenience sampling via the largest groups on Facebook focusing on individuals infected with COVID-19 and having long-term COVID-symptoms, COVID-19-related organizations, and primary healthcare centers in Stockholm, Sweden, treating post-COVID patients. The online announcement, including information about the study and a link to web-survey, was distributed via Facebook groups, Swedish COVID-organization, and the Karolinska Institute homepage. Participants could also access the web-survey by scanning Quick Response (QR) code provided during their visit at the primary health care centers. Thereafter, participants answered the survey in the online platform, Research Electronic Data Capture (REDCap), hosted locally at Karolinska institute^33,34^.

### Ethics approval

The study was approved by the Swedish national ethical board (dnr 2021-06617-01) and informed consent was obtained from all participants. All procedures utilized in collecting data for the current paper follow the ethical standards of the Helsinki Declaration of 1964 and subsequent amendments^35^.

### Measures

#### Contextual factors

Contextual factors in the current study consisted of personal factors including age, sex, education level, marital status, work status, and economic status and environmental factors including vaccinated against COVID-19, time of first infection, receiving treatment for post-COVID complaints, and hospitalization for COVID-19. Vaccinated against COVID-19 was measured with single item in which respondents indicated on a 4-point scale if they have received vaccine against COVID-19 (the first dose, the second dose, the third dose, vaccination not received). Time of first infection was measured with single item in which respondents were asked to report date of their infection/infections/positive COVID-19 test/tests (year-month). Receiving treatments for post-COVID complaints was measured using a single item in which respondents stated on a binary scale if they have/had received any treatment for their long-term symptoms after recovery from COVID-19 (yes, no). Hospitalization for COVID-19 was measured using a single item in which respondents stated on a binary scale if they have/had been hospitalized because of COVID-19 (yes, no).

#### Factors related to body functions and structures

Factors related to body functions and structures included infection with COVID-19, being a high-risk group for COVID-19, and severity of COVID-19 infection in the acute phase. Infection with COVID-19 was measured by a single item in which respondents stated on a 4-point scale if they have/had a confirmed COVID-19 infection supported by positive tests for COVID-19 virus (PCR) and/or positive rapid antigen test on a 4-point scale (I have had it one time, I have had it two times, I have had it more than two times, I believe I've had it, but have not had it confirmed). Being a high-risk group for COVID-19 was measured with a single item in which respondents stated on a binary scale (yes, no) if they have/had been at the high-risk group for COVID-19, such as high blood pressure, angina, stroke, heart disease, diabetes, cancer, smoking, respiratory diseases, and impaired immune system.

Severity of COVID-19 infection in the acute phase was measured with 15 items including fever, fatigue, cough, loss of smell and taste, difficulty breathing or shortness of breath, headache/migraine, aches or pain in body, diarrhoea, rash on skin, runny or blocked nose, nausea/vomiting, arrhythmia/palpitations, sore throat, cognitive difficulties such as memory and attention, and mental health problems such as sleep problems, depression and anxiety^36,37^. Here participants rated symptoms that they had at the beginning of the infection or infections and those the following 4 weeks on a 4-point scale (0 = no, 1 = mild, 2 = moderate, 3 = severe). The respondents’ answers to 15 symptoms of COVID-19 items were summed up to calculate the severity of COVID-19 infection in the acute phase (range 0–45, α = 0.77).

#### Post-COVID impairments

Post-COVID impairments were used as outcome variables and consisted of 54 items. This questionnaire was developed for the purpose of this study, based on previous questionnaires developed to examine long-term impacts of COVID-19 infection such as the ClinFIT COVID-19^29^ and the Functional Compass COVID-19 questionnaire^30^ and comprehensive literature review of the long-term effects of COVID-19^2,4,5,7,13,14^.

We first listed all common self-reported symptoms of post-COVID-19 condition in previous studies and then asked five experts (two psychologists, one physician, one occupation therapist, and one nurse) to review items and determine whether the items are relevant and also appropriate to be used as self-report measures. Based on the experts’ comments, a list of 54 items was finalized. Then, in the pilot phase, 10 individuals infected with COVID-19 supported by positive tests for COVID-19 virus (PCR) and still experiencing symptoms after the onset of infection were asked to answer the items. Then, cognitive interviews were conducted with participants in the pilot study to check whether the participants had any trouble to understand the items and how participants interpreted the items. For some items, a short description was added in order to provide more clarifications. In the final step, 54 items were adopted to the ICF as post-COVID impairments by two experts including 49 items covering b-categories and 5 items covering d-categories^28^. B-categories in ICF consist of impairments in body functions including 8 chapters. The current study sought to cover all eight chapters: 1. "Mental Functions" (14 codes); 2. "Sensory Functions and Pain" (14 codes); 3. "Voice and Speech Functions"(one code); 4. "Cardiovascular, Haematological, Immunological and Respiratory Systems" (5 codes); 5. "Function of the Digestive, Metabolic and Endocrine Systems" (8 codes); 6. "Genitourinary and Reproductive Functions" (one code); 7. “Neuromusculoskeletal and Movement-Related Functions” (3 codes); 8. "Functions of the Skin and Related Structures" (3 codes). Impairments related from chapter 3 to chapter 8 were considered as impairments in body system functions in the current study. D-categories in ICF consist of impairments in various actions and life areas. In the current study, 5 items were considered as impairments in activities and participation as results of the COVID-19 infection including difficulty taking care of yourself, impaired control of other diseases and drugs, and keep special diet, difficulties in doing housework, impaired work ability/study ability, and difficulty going to leisure activities. In this study, participants were asked to rate their difficulties/problems for each item on a 4-point scale (0 = no problem, 1 = mild problem, 2 = moderate problem, 3 = severe problem) (for a full description of the instrument see supplemental material).

Cronbach's alphas of the post-COVID impairments in the current study were *α* = 0.90 for impairments in mental functions, *α* = 0.88 for impairments in sensory functions and pain, *α* = 0.90 for impairments in body system functions, and *α* = 0.84 for impairments in activities and participation. The respondents’ answers to each sub-category of post-COVID impairments were summed up and divided by the number of items in order to calculate mean.

### Statistical analysis

Statistical analysis was performed using the IBM Statistical Software Package of Social Science (SPSS) version 26.0. Descriptive statistics was used for studying characteristics of the study sample. In addition, descriptive statistics of post-COVID impairments based on ICF categories was presented. For purposes of correlation and regression analyses, categorical variables were dichotomized for obtaining balanced distribution among variable categories; education (dichotomous: low education/high education), marital status (dichotomous: not relationship/in relationship), work status (dichotomous: not working/working), economic status (dichotomous: average and below average/above average), vaccinated against COVID-19 (dichotomous: not vaccinated/vaccinated), infected with COVID-19 (dichotomous: not confirmed COVID-19 infection/confirmed COVID-19 infection), and time of first infection (dichotomous: first and second wave of COVID-19 in Sweden/ during the year 2021 and 2022). A series of bivariate analyzes was performed to examine the associations between the related factors and post-COVID-19 impairments in order to identify primary predictors of the regression models via Pearson’s correlation. Candidate variables significantly associated with post-COVID impairment scores were regarded as potential explanatory variables for regression models. For predicting post-COVID outcomes (impairments in mental functions, impairments in sensory functions and pain, impairments in body system functions, and impairments in activities and participation), a series of hierarchical multiple linear regression analyses were performed. The aim was to examine the unique variance accounted for in the post-COVID impairment scores by the groups of variables and estimate the combined role of contextual factors and factors related to body functions and structures in accounting for variance in post-COVID impairments. Variables that were significantly associated with post-COVID impairment scores in the bivariate analyzes were included as three blocks in hierarchical multiple regression models predicting impairments after COVID-19 infection. Three blocks were entered in the following order: personal factors, environmental factors, and factors related to body functions and structures. We ran separate models for each outcome (impairments in mental functions, impairments in sensory functions and pain, impairments in body system functions, and impairments in activities and participation).

## Results

### Descriptive statistics for contextual factors

A total of 501 participants were included in the current study. Participants’ age ranged between 19 and 81 years old (M = 47.6, SD = 10.5 years old). The majority of the participants were female, had university/post graduate education level, working full or part time, and mainly was born in Sweden. The average time between reported COVID-19 infection and responding to the survey was 15.8 months (SD = 8.3) and the average length of hospital stay was 9.6 days in those who have/had been hospitalized because of COVID-19. Most of participants (85%) vaccinated against COVID-19 and among those 77% infected with COVID-19 for the first time before vaccination and the average time between reported first COVID-19 infection and received first dose of COVID-19 vaccine was 9.7 months (SD = 5.2). Contextual factors are presented in more detail in Table 1.

**Table 1:** Descriptive statistics of contextual factors including personal factors and environmental factors.

	N	%
Personal factors		
Sex		
Female	441	88
Male	60	12
Education level		
Compulsory school	14	2.8
Secondary	138	27.5
University/post graduate	349	69.7
Marital status		
Single	104	20.8
Married	209	42.7
In a relationship	144	28.7
Widowed/divorced/separated	44	8.8
Work status		
Working full time/part time	330	65.9
Unemployed	15	3
Retired	23	4.6
Parental leave	5	1
Sick leave	107	21.4
Unpaid work	8	1.6
Student	13	2.6
Self-rated economic status		
Below average	84	16.8
Average	243	48.5
Above average	174	34.7
Environmental factors		
Vaccinated against COVID-19		
Yes, one dose	21	4.2
Yes, two doses	156	31.1
Yes, three doses	241	48.1
No	83	16.6
Hospitalization for COVID-19		
Yes	51	12.5
No	358	87.5
Treatments for post-COVID complaints		
Yes	227	45.7
No	270	54.3

### Descriptive statistics for factors related to body functions and structures

Table 2 presents descriptive analysis of factors related to body functions and structures. The severity of COVID-19 infection in the acute phase ranged between 2 and 44 (M = 25.22, SD = 7.47). The most frequently reported symptoms were fatigue (98.6%), cognitive difficulties (90.7%), headache/migraine (90.6%), and aches or pain in body (88.9%).

**Table 2:** Descriptive statistics of factors related to body functions and structures.

	n	%
Infected with COVID-19		
Yes, one time	312	62.3
Yes, two times	103	20.6
Yes, more than two times	12	2.4
Yes, unconfirmed	74	14.8
Infected with COVID-19 for the first time		
First wave of COVID-19 in Sweden, spring 2020	159	38.7
Second wave of COVID-19 in Sweden, autumn 2020	87	21.2
During the year 2021	94	22.9
During the year 2022	71	17.3
Being a high-risk group for COVID-19		
Yes	98	45.7
No	397	54.3

### Descriptive statistics for post-COVID symptoms based on ICF categories

**Figure 1:**
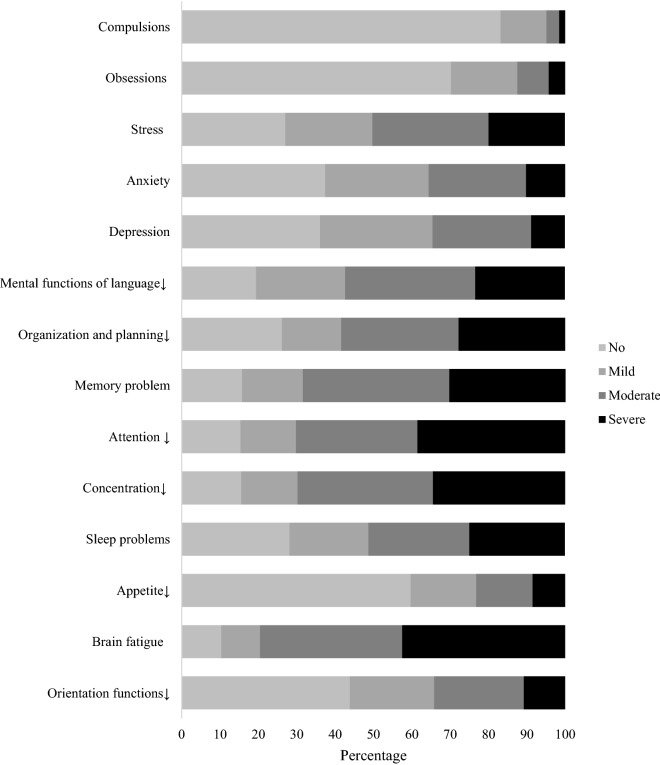
Proportions reporting impairments in mental functions.

Impairments in mental functions is presented in Fig. 1. Most commonly impaired mental functions reported by participants were sleep problem (71.9%), stress (73%), problems in organization and planning (73.9), language problems (80.6), memory problem (84.3%), concentration problem (84.5%), and attention problem (84.7%). Brain fatigue was reported as the most common impairment in the mental function section and was found in 89.7% participants, mostly moderate (37.1%) and severe (42.5%).

**Figure 2:**
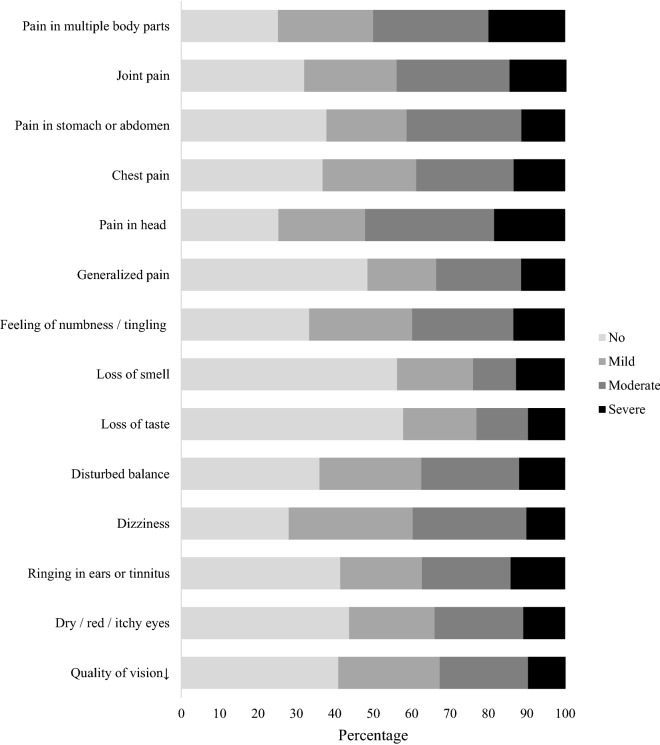
Proportions reporting impairments in sensory functions and pain.

Impairments in sensory functions and pain is presented in Fig. 2. Participants reported dizziness (72%) as the most impaired sensory function. Headache and pain in multi body parts were reported in 74.7% and 74.8% of participants, respectively.

**Figure 3:**
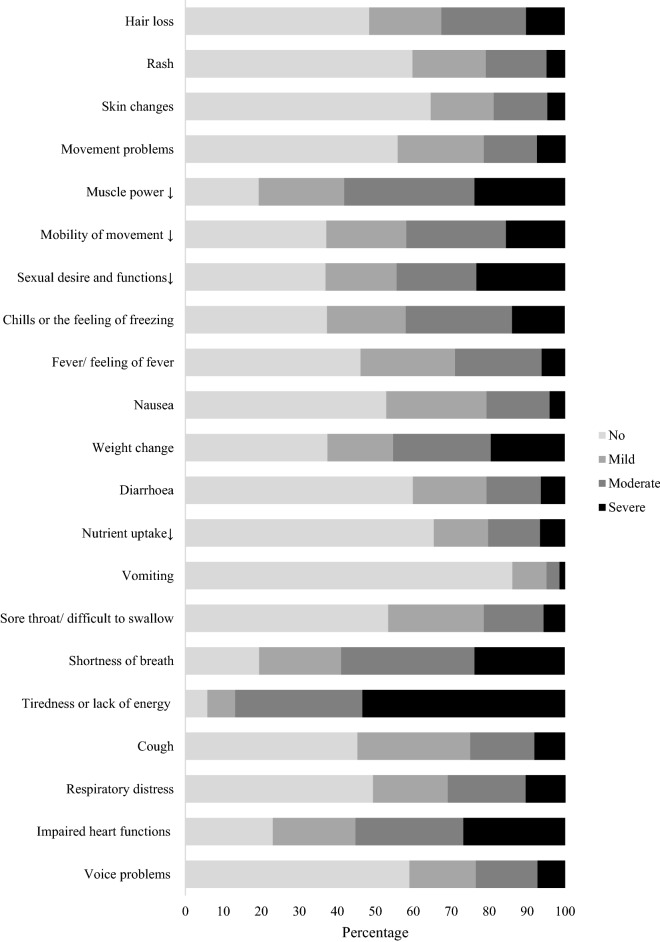
Proportions reporting impairments in body system functions.

Impairments in body system functions is presented in Fig. 3. Impaired heart function was reported in 77% of participants. Shortness of breath and decreased muscle power were reported in 80.6% and 80.7% of participants, respectively. The tiredness or lack of energy (fatiguability) was reported as the most impaired function in 94.2% participants, mostly moderate (33.5%) and severe (53.4%).

**Figure 4:**
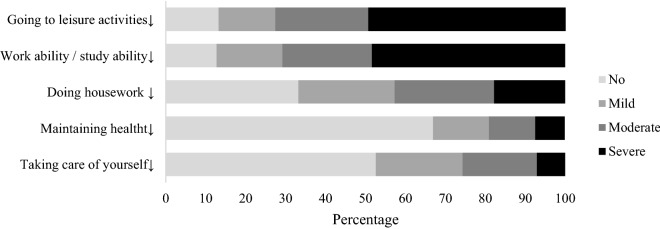
Proportions reporting impairments in activities and participation.

Impairments in activities and participation is presented in Fig. 4. The impaired work ability/study ability and difficulty going to leisure activities were reported as most impaired activities and participation in 87.3% and 86.8% of participants, respectively.

### Correlation between post-COVID impairments and contextual factors and factors related to body functions and structures

Table 3 presents correlations between the post-COVID impairments with contextual factors and factors related to body functions and structures. The severity of COVID-19 infection in the acute phase was the strongest correlate.

**Table 3:** Pearson correlations between related factors and post-COVID impairments.

Post-COVID impairments	Impairments in mental functions	Impairments in sensory functions and pain	Impairments in body system functions	Impairments in activities and participation
Personal factors				
Age	.085	.244**	.190**	.023
Sex	− .033	− .062	− .082	− .066
Education	− .098	− .166**	− .109*	− .018
Marital status	− .042	− .054	− .002	.002
Work status	− .181**	− .215**	− .172**	− .250**
Economic status	− .077	− .065	− .043	− .001
Environmental factors				
Time of first infection	− .203**	− .202**	− .245|**	− .204**
Vaccinated against COVID-19	− .098*	− .105*	− .151**	− .044
Been hospitalized	− .170**	− .177**	− .202**	− .188**
Treatment for post-COVID complaints	− .179**	− .111**	− .152**	− .307**
Factors related to body functions and structures				
Infection with COVID-19	− .036	− .070	− .028	− .069
Been high-risk group	− .088	− .129**	− .181**	− .074
Severity of COVID-19 infection	.534**	.604**	.626**	.352**

*p < .05, **p < .01.

### Regression analyses

Table 4 presents the hierarchical linear regression models for post-COVID impairments. The first block including the personal factors accounted for a small amount of the variance. Based on standardized regression coefficients, older age and lower educational level appeared as a relatively strong predictor of impairments in COVID-related sensory functions and pain. In addition, not working was a significant predictor for impairments in activities and participation. The second block, including environmental factors accounted for the moderate proportion of variance in the equations. Infected with COVID-19 for the first time in the first and second wave of COVID-19 in Sweden and not vaccinated against COVID-19 were the significant predictors for impairments in sensory functions and pain and impairments in body system functions. Not receiving treatment for post-COVID complaints was the significant predictor for impairments in activities and participation. The third block including factors related to body functions and structures was the main significant in the equations including 21.2% for impairments in mental function, 23.8% for impairments in sensory functions and pain, 25.7% for impairments body system functions and 8.6% for impairments in activities and participation. Severity of COVID-19 infection was clearly the strongest predictor.

**Table 4:** Hierarchical multiple regression analyses of personal factors, environmental factors, and factors related to body functions and structures and post-COVID impairments.

Predictor	Impairments in mental functions		Impairments in sensory functions and pain		Impairments in body system functions		Impairments in activities and participation	
	ΔR^2^	β	ΔR^2^	β	ΔR^2^	β	ΔR^2^	β
Block 1: Personal factors	.047**		.125**		.073**		.074**	
Age (range 19–81 years)		.019		.153**		.066		.031
Lower education		.100		.159**		.087		.033
Not working		.069		.095*		.067		.205**
Block 2: Environmental factors	.066**		.084**		.104**		.113**	
First infection in first and second wave		.097		.129**		.116*		.0.96
Been hospitalized		.008		.052		.091		.038
Not vaccinated		.094		.114*		.138**		.099
Not received treatments		.027		.007		.012		.214**
Block 3: Factors related to body functions and structures	.212**		.238**		.257**		.086**	
Been high-risk group		− .003		.018		.022		.005
Severity of COVID-19 infection		.484**		.514**		.534**		.307**
Total R^2^	**.32**		**.44**		**.44**		**.27**	

ΔR^2^ = R square change; β = Standardized regression coefficient.

*p < .05, **p < .01.

## Discussion

The primary aim of the current study was to map long-term impairments in individuals infected with COVID-19 by applying the framework of the World Health Organization’s International Classification of Functioning, Disability and Health^28^, extending the previous studies^29,30^. To further enhance the knowledge about post-COVID impairments, the secondary aim was to study the influence of contextual factors (personal factors and environmental factors) and factors related to body functions and structures on impairments following COVID-19 infection. The results revealed that post-COVID impairments reported more frequently and the most common one was fatigue. In addition, the strongest risk factor for post-COVID impairments was severity of COVID-19 infection in the acute phase.

The majority of participants in the present study experienced mild COVID-19 infection and they were not a high-risk group for COVID-19. Furthermore, the major proportion of participants reported that they have/had been infected with COVID-19 supported by tests for COVID-19 virus (PCR) and/or rapid antigen test at least one time and only 15% reported that they believe they' have had COVID-19 but have not had it confirmed.

In this study, 54 items were considered as impairments due to COVID-19 infection/infections adapted to ICF. We found on a whole that post-COVID impairments occurred very frequently. In that, 96% of the study sample reported at least one moderate-to-severe impairment due to COVID-19 infection. The proportions of post-COVID impairments in our sample were higher than the prevalence of the post-acute symptoms reported in previous studies^3,38–41^. One possible explanation for these findings lies in our targeted convenience sampling. Another possible explanation of these results is the timing of data collection and assessment methods. As expected, fatigue was in general the most common reported persistent symptom. This finding is in line with previous studies showing that fatigue is a primary complaint, and it is the commonest complaint in a large group of COVID-19 survivors^42–48^. There were two items related to fatigue in our study, brain fatigue categorized under impairments in mental functions and physical fatigue categorized under impairments in body system functions. Moderate-to-severe brain fatigue and tiredness or lack of energy (fatiguability) were reported by the majority of the participants. Consequently, eight in ten reported that their activities and participation were impaired. In that, the most common impaired life areas were impaired work ability/ study ability and difficulty going to leisure activities. These findings are in line with previous study in Sweden showing that work, social, and home life were disrupted in a considerable proportion of individuals 8 months after mild COVID-19 infection^49^.

In our study, the degree of impairments varied and higher levels of post-COVID impairments were associated with older age, lower education level, not working, infected for first time during the first and second pandemic waves in Sweden, not vaccinated against COVID-19, hospitalization due to COVID-19, not receiving treatment for post-COVID complaints, being at a high-risk group for COVID-19 infection, and higher severity of COVID-19 infection in the acute phase. However, prior severity of COVID-19 infection was the strongest correlate.

Personal factors contributed unique variance in all of the four post-COVID impairments. Looking at the role of the separate personal factors revealed that older people are more vulnerable to impairments in sensory functions and pain. In the other words, older age is a risk factor for post-COVID impairments, at least impairments in sensory functions and pain. Results related to association between age and post-COVID symptoms are inconsistent, reporting a positive association^39,41^, a negative association^40^, and no association^50^. In addition, lower educational level was found to be associated with higher risk of post-COVID impairments in the current study. The previous findings regarding education and long-term symptoms of COVID-19 are also inconsistent. For example, the results from a national registry-based study in Sweden found no association between education and long COVID^51^. However, another study showed that lower levels of education are a risk to develop persistent symptom after COVID-19 infection^52^. Not working including unemployed, retired, parental leave, sick leave, unpaid work, and student was found to be a strong predictor for impairments in activities and participation. This finding has been interpreted as for example being on sick leave typically could disrupt family, working, and social life^53^. The reduced work ability was reported 7 months after the onset of disease^43^. Taken together, it can be concluded that contribution of personal factors as a risk of developing post-COVID impairments is not clear.

In this study, environmental factors were analyzed. Time of first infection, being hospitalized, and vaccinated against COVID-19 contributed unique variance in the impairments in sensory functions and pain and impairments in body system functions. In addition, receiving treatments for post-COVID complaints contributed unique variance in the impairments in activities and participation. Looking at the importance of the individual variables within the set, infected with COVID-19 for the first time during the first and second pandemic waves in Sweden (the spring and autumn of 2020) contributed to the explained variances in impairments in sensory functions and pain and impairments in body system functions. It is well established that early variants of SARS-CoV-2 in prior waves appeared to cause more severe acute illness than later variants^54,55^. Moreover, recent studies showed that the prevalence and profile associated with post-COVID condition vary across different SARS-CoV-2 variants^55–57^. It is therefore reasonable to conclude that those infected in the early waves of pandemic in Sweden continue to be a vulnerable group to have more severe complaints due to COVID-19 infection and in need of support.

Our findings showed that rates of post-COVID impairments were considerably higher in not vaccinated group compared to vaccinated group. These differences were more considerable between non-vaccinated participants and those who received three doses. This finding is in line with previous studies which showed that the risk of post-COVID complaints differed as function of vaccinate status. In that, post-COVID complaints are more common in non-vaccinated individuals compared to vaccinated individuals^56,58–64^. In addition, studies show that those who received double immunization have a lower risk of post-COVID impairments^22,54^. However, recent findings showed that the time of vaccination may be associated with post-COVID complaints. In that, vaccination before COVID-19 infection could reduce risk of post-COVID complaints but impact of vaccine in people with post-COVID complaints is still unclear^65^. In the current study, being hospitalized did not contribute to the explained variances in post-COVID impairments. However, several studies showed that rates of persistent symptoms are higher in individuals who have been hospitalized because of COVID-19 compared with matched control group^2,66–68^. One explanation for this finding relies on that the majority of participants in the current study experienced mild COVID-19 infection and only 12.5% of participants have/had been hospitalization because of COVID-19 infection.

Nearly half of participants in the current study received post-COVID-related interventions. The results revealed that receiving any type of treatment options for post-COVID complaints could benefit activities and participation. It is therefore important to provide facilities for individuals with post-COVID impairments to access interventions in the health care services. In this study, the best single predictor of the post-COVID impairments was factors related to body functions and structures, accounting for 21.2%, 23.8%, 25.7%, and 8.6% of variance in impairments in mental functions, sensory functions and pain, body system functions, and activities and participation, respectively. These findings are perhaps expected. Looking at the importance of the separate variables within the set, prior severity of COVID-19 infection appeared as the strongest predictor of post-COVID impairments. This finding is in line with previous studies which mentioned that the severity of COVID-19 infection was correlated with the severity of post-COVID-19 manifestations^3,39,42,69–72^ and is a strong risk factor for experiencing persistent symptoms^38,41^.

### Strengths and limitations

There are several strengths in our study. From a methodological point of view, using the ICF classification and including many variables and measures provides extensive information regarding the understanding of post-COVID symptoms in relation to other symptoms and variables. This further enhances our knowledge regarding contextual factors and factors related to body functions and structures relevant for clinicians, policymakers, and patients during screening and treatment. Furthermore, the fact that researchers, at an early stage together with clinicians, were involved in the selection of factors of interest further enhances the validity of the study. The endorsement of post-COVID impairments in our sample indicate that we managed to collect data from our target population. This probably has to do with the different ways of recruitment in the study. Also, our plan for analyzing data in several steps provided clarity to the final hierarchical regression and ensured relevant outcomes.

However, there are several limitations associated with this study that should be considered when interpreting the findings. First, the main limitation of the study is that only self-reported data were obtained. It might have influenced the accuracy of reporting symptoms and also severity of symptoms due to recall bias. Second, all data are obtained from respondents that were fluent in Swedish and had access to internet which weakens the ecological validity. Third, respondents in the current study had good enough abilities mentally and physically to answer the web-survey. Therefore, there is a risk that we have missed individuals who have had severe COVID-19 infection and have been very sick. Fourth, the small number of people included in the survey, which may lead to biased or inaccurate conclusions. Fifth, the timing of COVID-19 infection is crucial for understanding the post-COVID impairments. However, the time of infection of COVID-19 was not considered in the population under investigation in the current study. All these limitations may influence the generalizability of the study results.

### Future research

More longitudinal studies are needed to identify how post COVID-19 impairments change over time. Also, qualitative studies focusing on the subjective experience of a patient are needed in order to wholly understand the implications of post COVID-19 impairments. Further validation of the classification model is needed to ascertain that the symptoms used to describe post COVID-19 impairments are meaningful. Finally, the role of viral persistence in post-COVID impairments is still under investigation and further research is needed.

## Conclusion

The study mapped possible post-COVID impairments in individuals infected with COVID-19 by applying the framework of the ICF and found that contextual factors and factors related to body functions and structures contributed to post-COVID-19 impairments. Post-COVID impairments reported more frequently and the most common one was fatigue. COVID-19 severity in the acute phase explained a great deal of variance for the impairments. The knowledge gained can be used to tailor strategies to decrease the burden of symptoms and thus have a serious impact on lessening the aftermath of the epidemic.

## Supplementary Information

Supplementary Information.

## Data Availability

Data are available upon reasonable request from the corresponding author.
