# Supplementary material for: Impairments following COVID-19 infection: manifestations and investigations of related factors
Source: Sci Rep. 2023 Apr 21;13:6564. doi: 10.1038/s41598-023-33810-y (PMC10119844; doi:10.1038/s41598-023-33810-y)
Supplement: Supplementary file 1 — Supplementary Information. [file 41598_2023_33810_MOESM1_ESM.pdf]

# Impairments following COVID-19 infection: Manifestations and investigations of related factors

Farzaneh Badinlou, David Forsström, Markus Jansson-Fröjmark, Tamar Abzhandadze, & Tobias Lundgren

Supplemental material. Items of post-COVID impairments according to ICF

| ICF code | Item |
|---|---|
| **Impairments in mental functions** | |
| b114 | Impaired orientation functions such as difficulty of knowing and ascertaining one's relation to self, to others, to time and to one's surroundings |
| b 1300 | Brain fatigue |
| b 1302 | Lack of appetite |
| b 134 | Sleep problems |
| b 140 | Concentration problem such as difficulty of focusing on a task or topic |
| b 140 | Attention problem such as difficulty of focusing on one part of an experience and ignoring others or focusing on several things |
| b 144 | Memory problem |
| b 1641 | Impaired organization and planning such as difficulty of coordinating parts into a whole) |
| b 167 | Impaired mental functions of language such as difficulty of recognizing and using signs, symbols and other components of a language |
| b 152 | Depression |
| b 152 | Worry/ anxiety |
| b 152 | Stress |
| b 160 | Obsessions such as unwanted thoughts and fears |
| b 160 | Compulsions such as you feel compelled to do certain things |
| **Impairments in sensory functions and pain** | |
| b 2102 | Impaired quality of vision |
| b 220 | Dry / red / itchy eyes |
| b 2400 | Ringing in ears or tinnitus |
| b 2401 | Dizziness |
| b 235 | Disturbed balance |
| b 250 | Loss of taste |
| b 255 | Loss of smell |
| b 265 | Feeling of numbness / tingling |
| b 2800 | Generalized pain |
| b 28010 | Pain in head |
| b 28011 | Chest pain |
| b 28012 | Pain in stomach or abdomen |
| b 28016 | Joint pain |
| b 2802 | Pain in multiple body parts |
| **Impairments in body system functions** | |
| b 310 | Voice problems |
| b 410 | Impaired heart functions |
| b 440 | Respiratory distress |
| b 450 | Cough |
| b 4552 | Tiredness or lack of energy (Fatiguability) |
| b 460 | Shortness of breath |
| b 5105 | Sore throat/ difficult to swallow |
| b 5106 | Vomiting |
| b 515 | Impaired nutrient uptake |
| b 525 | Diarrhoea |

| | |
|---|---|
| b 530 | Weight change |
| b 5350 | Nausea |
| b 5500 | Fever/ feeling of fever |
| b 5500 | Chills or the feeling of freezing |
| b 640 | Impaired sexual desire and functions |
| b 710 | Impaired mobility such as difficulty to move knee, ankle, hands, feet. |
| b 730 | Decreased muscle power |
| b 760 | Impaired control over and coordination of movements |
| b 810 | Skin changes |
| b 840 | Rash/ itching |
| b 850 | Hair loss |
| **Impairments in activities and participation** | |
| d 5 | Difficulty taking care of yourself |
| d 5 | Impaired control of other diseases and drugs, keep special diet |
| d 6 | Difficulties in doing housework |
| d 8 | Impaired work ability / study ability |
| d 9 | Difficulty being to leisure activities |
